# “I feel like there’s going to have to be catastrophe before I am seen:..and that is going to be devastating”: Visibility, recognition, and medical silencing in eating disorders and disordered eating

**DOI:** 10.1371/journal.pmen.0000634

**Published:** 2026-06-17

**Authors:** Hannah Healey, Claudia Susana Caxaj, Elysée Nouvet

**Affiliations:** 1 Health and Rehabilitation Sciences, Western University, London, Ontario, Canada; 2 Arthur Labatt Family School of Nursing, Faculty of Health Sciences, Western University, London, Ontario, Canada; 3 School of Health Studies, Western University, London, Ontario, Canada; Assiut University, EGYPT

## Abstract

Eating disorders (EDs) and disordered eating behaviours (DEBs) are major global health concerns that remain under-recognized and under-treated. In addition to structural barriers, individuals encounter symbolic and epistemic obstacles, including failures of recognition, diagnostic exclusion, and medical silencing. These processes shape who is legitimized as having EDs/DEBs within biomedical systems and society. To examine how such gaps are produced and navigated, we employ a narrative intersectional methodology. Although analysis was grounded in inductive coding, interpretations were informed by lived-experience alongside narrative and intersectional commitments. We present the overarching theme Who Gets Seen: Recognition and Visibility in EDs/DEBs. Three subthemes emerged: *Struggles for Legitimacy*, *Being Seen, Misread, or Overlooked*, and *Navigating Self-Recognition*. Across themes, participants described barriers to care, recognition, and healing, illustrating how individuals negotiate norms of being seen by family, friends, healthcare providers, and themselves. Findings highlight persistent inequities in recognition and visibility that leave many individuals unnoticed and undertreated. These dynamics reflect epistemic injustice, where credibility and lived realities are undermined within medical systems. Advancing epistemic justice requires reimagining what EDs/DEBs look like and who is considered deserving of recognition and care.

## Introduction

An estimated 70 million people worldwide are affected by eating disorders (EDs) [1,2]. EDs are serious global health concerns associated with high morbidity and mortality [3]. Their impacts extend beyond individuals to families and caregivers [4,5], and healthcare providers who experience unique stresses and harms in delivering care [6]. Disordered eating behaviours (DEBs) similarly cause distress, reduce quality of life, and increase risk for EDs [7]. Despite rising demand for treatment [8], EDs remain underdiagnosed and undertreated [9,10], while DEBs are rarely measured, leaving many patients struggling in silence and without recognition.

In Canada and internationally, physical access to ED/DEBs treatment remains persistently constrained. Some of these well-documented barriers include: prolonged wait times for assessment and treatment [11,12], substantial regional variability in the availability and intensity of publicly funded services [13], and significant discontinuities in care during transition from youth to adult services [14,15]. Geographic inequities also shape access, as specialized ED/DEB services remain disproportionately concentrated in large urban centres [16], often requiring extensive time commitments that are incompatible with employment, caregiving responsibilities, and other forms of paid and unpaid labour. While virtual treatment modalities have expanded in recent years, these options may themselves reproduce inequities, particularly for individuals in rural or remote regions with limited internet infrastructure [17]. These barriers illustrate how physical accessibility to ED/DEB care is structured by broader health systems organization, geography, and social conditions rather than solely on service availability.

Beyond physical and other overt dimensions of accessibility, achieving equitable care necessitates attention to less visible—and often overlooked—aspects of accessibility. Symbolic and epistemic access—recognition, diagnosis, and visibility as someone struggling with illness—are equally vital [18–20]. Without recognition, physical access alone cannot ensure equitable care. The ability to see oneself—or be seen—as having a “legitimate” ED/DEB is central to inclusive and just treatment pathways.

Intersectionality [21,22] illuminates how identities (e.g., race, body size, gender, class, sexuality) and contextual factors (e.g., place, timing, service availability) intersect, shaping recognition. These intersections can produce both epistemic privilege and violence [20], contributing to medical silencing [19]. Recognition—self, medical, or social—remains uneven, and distorted by stigma, narrow diagnostic categories, and overemphasis on BMI and “severity,” privileging biomedical models while obscuring lived-experience.

EDs/DEBs have long been framed through the “skinny, white, affluent girl” (SWAG) stereotype [23].This narrative shapes who is seen as having an ED and who remains invisible, despite evidence that EDs affect diverse populations [24–26]. This narrow conception is often reinforced in both healthcare and research [27,28], leaving many struggling in silence—or unaware of their condition—because frameworks to recognize their experiences as disordered are absent.

As Fricker [20] notes, prejudice often operates through stereotypes embedded in the collective imagination, shaping recognition beneath conscious scrutiny. Applied to EDs/DEBs, such stereotypes erase visibility for those struggling—whether to themselves, providers, or family—while shaping broader social expectations of what EDs/DEBs ‘should’ look like.

Despite their widespread use, weight and Body Mass Index (BMI) are neither inherently accurate nor meaningful indicators of health, particularly in EDs/DEBs. Individuals in non-normative bodies may experience significant distress and disordered eating yet remain unrecognized when weight does not align with clinical expectations. Although framed as objective, weight- and BMI-centered tools are arbitrary and historically rooted in white, male populations, rendering them insensitive to diversity and lived-experience [29,30].

In the context of ED/DEBs specifically, weight and BMI operate as epistemic and structural barriers to recognition, treatment, and recovery [31]. BMI functions less as a health measure than as a legitimizing tool that governs who is seen as “sick enough” to receive care [29,30]. Individuals with lived-experience describe BMI and weight thresholds as gatekeeping mechanisms that invalidate suffering and obstruct access [32]. Weight stigma—embedded within biomedical discourse—further compounds harm, reinforcing shame and discrediting patient testimony [33]. As McEntee and colleagues [33] argue, weight loss is neither a prerequisite for health nor an appropriate treatment target. Importantly, this stigma is a global issue, with adolescents in Brazil, South Africa, and Indonesia also reporting weight-related discrimination [34], underscoring weight stigma as a global health concern [35]. Beyond BMI, marginalization of ED/DEB presentations outside anorexia nervosa (AN) or bulimia nervosa (BN) is well-documented [27,31]. Those diagnosed with other disorders (e.g., BED, OSFED, ARFID) often face exclusion due to weight-centric criteria [36]. Although AN and BN remain serious concerns, modelling treatment eligibility predominantly around these presentations leaves other unseen.Individuals with atypical anorexia face risks comparable to AN but are often dismissed without a low BMI [31]. These patterns underscore the need for weight-neutral, trauma-informed care.

These barriers highlight the need for research that moves beyond documenting access deficits to examining how individuals with EDs/DEBs experience, interpret, and navigate care across contexts. Such understanding is essential for informing policies and practices attentive to lived constraints and intersecting marginalization shaping diagnosis and treatment. Addressing these gaps, this study explores the experiences of individuals marginalized in recognition, diagnosis, and recovery, with attention to barriers and facilitators influencing visibility, recognition, and lived-experience.

### The present study

Fricker [20] defines epistemic violence as harm that undermines one’s capacity as a knower, limiting participation in meaning-making, testimony, and credibility. She distinguishes two forms: testimonial injustice—the devaluation of testimony due to prejudice—and hermeneutic injustice, which arises from gaps in shared interpretive resources (e.g., diagnostic categories, normative frameworks). Rooted in power and the privileging of certain bodies and narratives, these dynamics are especially salient in health contexts where patients’ lived knowledge is routinely discounted.

Building on this, Dhairyawan [19] describes medical silencing, whereby patients’ reports of their bodies, symptoms, or suffering are dismissed. Such silencing fosters mistrust and discourages care-seeking [18]. In DEB/ED contexts, individuals are often stereotyped as unreliable or “non-compliant,” their testimonies treated with suspicion [37], often resulting in disengagement and treatment dropout.

Drawing on epistemic injustice and medical silencing scholarship [18–20] and informed by intersectionality [21,22,38], this narrative-based study examines how these processes shape recognition, diagnosis, and access to care across intersecting identities.

## Materials and methods

### Positionality

The primary researcher (HH) identifies as female, and brought both professional and lived experience with DEBs and EDs to this work, which informed the study design, data collection, and analytic approach. Details of HH’s lived experience are published in more depth elsewhere [39]. HH is a PhD Candidate, and was a practicing Research Coordinator at the time of conducting this study. Reflexive practices such as memoing and journaling were employed throughout the research process to critically attend to how this dual positioning shaped interpretation and analytic decision-making. The primary researcher (HH) also brought eight years of experience conducting qualitative health research, which informed methodological decision-making and analytic depth. This work is therefore deeply personal, and foregrounds lived-expertise as a source of analytic insight.

The co-authors (SC, EN) are senior qualitative researchers and associate professors with PhDs, both with established programs of research, who provided conceptual guidance, study oversight, and critical input throughout the research process. Both identify as female. Collectively, these positionalities guided rather than constrained the work, shaping how meaning-making and knowledge generation unfolded. The findings reflect an analytic process marked by extended data immersion, iterative reflexivity, and a sustained commitment to amplifying participants’ voices [40,41].

### Methodological approach

The overarching methodological approach was narrative-based, chosen for its capacity to capture how individuals co-construct meaning through lived-experience [42,43]. Grounded in intersectionality as both a theoretical lens and guiding paradigm [21,22,38,44], this work operationalized its principles throughout the research process, as elaborated in a forthcoming manuscript. Ontologically, categories such as race, gender, class, and ability are viewed as mutually constitutive rather than discrete [38,44,45]. Epistemologically, critical knowledge is understood to emerge from lived-experience, particularly through accounts of power, privilege, marginalization, and resistance. Intersectionality thus operates as both theory and paradigm, informing—and informed by—narrative approaches that illuminate complex lived realities. This study followed consolidated criteria for reporting qualitative research [46]; a COREQ checklist is provided in the supplemental materials (S1 File).

### Ethics

Ethics approval was obtained from Western University’s Research Ethics Board (**Project ID:** 123540). Written informed consent was obtained by the primary author, with willingness to participate reconfirmed verbally before each interview. All participant quotations have been de-identified, and pseudonyms are used throughout.

### Community partnership

This study was conducted in partnership with a lived-expertise–led, community-based not-for-profit organization (Body Brave) providing low-barrier support for individuals with eating disorders across Canada. Body Brave offers free or publicly funded virtual services, including online resources, treatment groups, and clinical intake support for adults. Programs are developed in consultation with individuals with lived experience. The research—from design to knowledge mobilization—was also guided by a Lived-Experience Advisory Committee made up of individuals with lived or living experience with EDs/DEBs.

### Recruitment

Twenty-three participants with self-identified EDs/DEBs were recruited via our community partner’s online newsletter which is distributed by email to anyone who has signed up to receive messaging from Body Brave upon their registration with the organization. Recruitment commenced June 24^th^, 2024, and concluded August 10^th^, 2024. Inclusive recruitment materials were used to engage individuals without formal diagnoses and those often underrepresented in treatment. Researchers had no prior relationships with participants, and eligibility criteria included age ≥ 18 and English proficiency.

To document diverse experiences of recognition and visibility in EDs/DEBs, we employed purposive maximum variation sampling [47] to capture patterns across varied lived experiences. Participants first completed a demographic survey administered via institutional Qualtrics, designed in line with best practices for equitable demographic data collection [48]. The survey collected information on age, ability, race, ethnicity, income, sexuality, gender, and employment status, and included an open-ended text box for additional self-identified characteristics. Based on rolling survey responses, participants were purposively selected to ensure maximal variation. Of 52 respondents, 23 were selected for interviews and subsequent focus groups; the remaining 29 were notified that they were not selected and thanked for their time. The aim was not broad representativeness, but diversity across intersecting identities and social locations. All 23 participants proceeded through all study procedures, and we did not have anyone decline to participate or drop out during the progression of the study.

This qualitative study did not aim for generalizability, but to describe diverse lived-experiences [49,50]. Sample size was therefore not determined by saturation. Instead, decisions were guided by five considerations related to information power: study aim, sample specificity, use of theory, quality of dialogue, and analytic strategy [51]. The sample was intentionally broad; although similar themes emerged, a maximal variation approach ensured they reflected diverse experiences and positionalities. Recruitment concluded after 23 interviews based on these criteria and pragmatic considerations, including research team capacity, assessed by all authors.

### Participants

Guided by an intersectional paradigm, in this study we attended to how multiple intersecting social locations shape participants’ experiences, while also recognizing that detailed demographic reporting can increase risks of identification in qualitative research*.* Accordingly, demographic information is presented below in a deliberately aggregated manner to balance analytic transparency with participant privacy.

Of the 23 participants who took part in this study, all self-identified as belonging to an equity-deserving group (n = 21) or as a member of an under-represented group (e.g., men; n = 2). Participants reported a range of racial and ethnic identities, including Black (n = 1), Chinese (n = 3), Latin American (n = 4), Middle Eastern (n = 1), Métis (n = 1), South Asian (n = 2), White (n = 12), and one participant selected ‘Other’ self-identifying as African (n = 1).

The majority of participants (n = 15) self-identified as having a disability or chronic condition. Reported conditions included learning disabilities (n = 6), mobility-related disabilities (n = 4), speech-related disabilities (n = 1), mental health conditions (n = 4), and being blind or having low vision (n = 3). Four participants selected “other” and self-described their condition in an open text field, with examples including long COVID and chronic pain. Participants identified across a range of gender identities, including female (n = 15), non-binary (n = 4), male (n = 4), and transgender (n = 2). Many participants described challenges situated at the intersection of these—and other—identities. Additional demographic information is provided in the supporting material (S1 Table).

### Data collection

Three data collection methods were used: a demographic survey, semi-structured interviews, and member-reflection focus groups. The demographic survey (June–August 2024) informed maximal variation sampling and provided contextual information used to situate participants’ narratives (see https://osf.io/kgpsx). Demographic discussions also served as an entry point for interviews, supporting early reflection on intersectional identities and their relevance to experiences of EDs/DEBs.

Semi-structured interviews were conducted by the primary author (HH) between June and September 2024 using a collaboratively developed, pilot tested, guide (see https://osf.io/kgpsx). Each participant took part in one interview. Prior to each interview, the first author (HH) disclosed to participants that she has personal lived experience with an ED, including experiences of both accessing and being denied care. This disclosure was intended to establish a sense of shared experience and positionality. HH was intentional in keeping this disclosure brief, so as not to invoke particular imagery of what an ED or DEBs are ‘supposed’ to look like, recognizing the diversity of presentations and experiences that exist. Following demographic reflection at the outset, interviews focused on participants’ narrative accounts of ED/DEB experiences, identity-related influences, patterns of recognition and exclusion, treatment access, and interactions with healthcare services. Interviews were conducted with only the interviewer (HH) and participant present via Western’s institutional Zoom (25–120 minutes (M = 66 min)). They were audio-recorded, supported by reflexive field notes, transcribed verbatim, and de-identified. The interview design centred a narrative methodology [42,43], recognizing participants’ accounts as situated, relational, and shaped by intersecting social positions.

Transcripts were not returned to participants for member checking. Interviews were lengthy, and reviewing full transcripts would have required a substantial additional time commitment. Because the study did not have external funding and therefore lacked the capacity to compensate participants for this additional work, returning transcripts was considered likely to impose an undue burden on participants. In line with ethical considerations around participant burden, transcripts were therefore not circulated for review. Participants did have the opportunity to provide feedback on preliminary themes during the member reflection focus groups, which are described in greater detail below.

To enhance reflexivity and participant involvement, three member-reflection focus groups (FG1–FG3; n = 3–4) were conducted by the primary author (HH) between June and August 2025 over Western’s Institutional Zoom. These sessions (90–105 minutes) provided space for participants to engage with and refine preliminary thematic interpretations and were recorded, transcribed, and de-identified. The first author (HH) presented emergent thematic areas to participants and facilitated discussion using a semi-structured focus group guide (see *https://osf.io/kgpsx*), collaboratively developed and piloted by the research team (HH, SC, EN), to prompt critical reflection on the themes. Reflexive field notes were taken by the first author (HH) before and after each of the three focus groups. Participants were explicitly invited to identify anything they felt did not resonate or fit within the themes; no such concerns were raised. We nonetheless remain reflexively aware that power dynamics inherent to research relationships may have shaped participants’ comfort in voicing dissenting perspectives, regardless of the steps taken to mitigate such influences. Focus group contributions are identified in the Results section using focus group (FG 1, FG 2, or FG 3) labels and pseudonyms.

### Analysis

De-identified focus group and interview transcripts were uploaded to NVivo 14 (QRS International) for analysis. A narrative-informed approach to reflexive thematic analysis guided the interpretation of interviews and member-reflection focus groups [40–42,52]. This approach foregrounds co-constructed narratives and allows for the exploration of complexity within participants’ accounts [40–42,52]. All transcripts were inductively coded by the primary author (HH), with codes generated directly from the transcripts, rather than determined a-priori. A preliminary codebook was developed and iteratively refined through ongoing discussion between HH and EN. As analysis progressed from codes to themes, all authors (HH, EN, SC) engaged in discussions regarding coding decisions and thematic development to support reflexivity and analytic coherence. To strengthen credibility, themes and illustrative quotes were also presented to and reviewed by the lived-experience advisory committee and discussed in member-reflection focus groups, where participants validated, extended, or challenged interpretations. Insights from these discussions were thematically analyzed [40–42,52] and integrated into the evolving codebook.

## Results

### Overview of thematic findings

We report here on a key thematic finding resulting from participants’ accounts and our analysis—*Who Gets Seen: Recognition and Visibility in DEBs/EDs.* Within this theme, we identified three subthemes: *“You have to present a certain way…”: Struggles for Legitimacy*, *“Nobody was worried about me…”: Being Seen, Misread, or Overlooked,* and *“I just thought that it was me being fat and lazy…”: Navigating Self-Recognition*. Together, these subthemes contextualize how those struggling with DEBs/EDs navigate multi-level recognition—and subsequent access to care—at sociocultural, relational, institutional, and personal levels.

### “*You have to present a certain way*…”: Struggles for legitimacy

Participants described feeling excluded and de-legitimized in DEB/ED treatment, through dynamics that were both systemic and individual. Their accounts centred on two recurring storylines: *Gatekeeping Mechanisms and Markers of (So-Called) Severity,* and *Coming up Against Medical Categories and Labels.* Through varied experiences, participants recounted a shared storyline of having to jump through countless hoops and manage how they appear in order to be recognized and taken seriously in care. As Vivian put it, *“you have to present a certain way physically to get the standard of care that you deserve.”* Others noted that even this form of conformity was not available to everyone—those whose bodies or identities did not align with normative expectations were often denied recognition entirely.

Gatekeeping Mechanisms and Markers of (So-Called) Severity*.* Participants described how weight, BMI, and other physical indicators operated as over-relied upon markers of severity within DEB/ED care. While weight and BMI emerged most prominently in their narratives, additional indicators—particularly those deemed more “visible,” whether through appearance or through ostensibly objective measures (e.g., abnormal laboratory results or cardiovascular complications)—were also experienced as benchmarks against which treatment legitimacy was assessed. Collectively, these markers functioned as institutionalized proxies for severity, reinforcing the notion that individuals who could visibly or quantifiably demonstrate their illness were considered more credible and deserving of care.

*It still kind of feels like in terms of screening criteria that because I’m not at a critical kind of hospitalization sort of situation…that treatment isn’t sort of available to me.”-Zara*

Within treatment, weight and BMI continue to serve as dominant reference points for both access to and participation in services. A less acknowledged dimension of this emphasis is the extent to which such metrics are internalized by individuals themselves, shaping perceptions of legitimacy, illness severity, and treatment deservingness. Participants frequently reflected on how weight and BMI operated within their treatment experiences and how they understood or positioned themselves in relation to these measures. Vivian, for instance, underscored how presenting at a lower weight functioned as a proxy for the level of support she received.

*“Presenting a certain way was like a proxy for the severity of your illness and how much you need help… especially if you present even like a normal weight or maybe even overweight, like, it’s just a lot harder to be taken seriously…”*

Participants also demonstrated an acute awareness that bodies not aligning with dominant, stereotypical representations of eating disorders were frequently rendered invisible—to family, friends, treatment providers, and, at times, even to themselves. Kimberly described this experience as “flying under the radar,” attributing this to how her body was perceived by others: *“[my eating disorder] is hidden in terms of like the extremes of it, and having a body that is not visible, not visibly denied…makes me fly under the radar.”*

Such accounts highlighted how epistemic authority within (and outside of) clinical settings is often conferred through more visible (often weight-based) quantitative markers of illness. Participants described internalizing these judgements, sometimes experiencing shame and embarrassment both when comparing themselves with peers in treatment, and when encountering care providers who—by way of body language, tone, or explicit comments—communicated that they were not at a sufficiently low weight or level of severity to justify intervention. These interactions reinforced broader discourses of legitimacy in DEB/ED care, and contributed to a pervasive sense of being undeserving, insufficiently ill, or an undue burden on already limited resources.

*“I never felt like I got to like a low enough weight … compared to everyone else... when my doctor or my psychologist would want to put in a referral and I’d be like, my weight’s not low enough…they’re going to laugh at this like and say I don’t need treatment.” -Ava*

This overemphasis on weight also translated into tangible gaps in treatment, creating circumstances in which participants consequently fell through the cracks—deemed too low in weight to qualify for certain programs, yet too high for others.

***“*** *So, my weight being, like, it, a bit higher, like not at the dangerously low levels, it means I can’t get into, yeah, the inpatient treatment that I need, but my weight is too low for me to get the outpatient treatment here that I need… I’m not sick enough to get some treatment, but I’m too sick to get other treatment… I’m helpless and hopeless.” -Reese*

Several participants described the direct harms that flow from an overemphasis on weight: systems-level requirements that someone appear emaciated or acutely ill to be deemed “worthy” of care. These eligibility constraints, they said, provoke despair and discourage help-seeking—forcing people with DEBs/EDs to deteriorate before they can access treatment. For example, Ava observed, *“it just makes it harder hearing those comments and feeling like, okay, I’m not valid of treatment. Like if I’m going to go like I need to be sick enough.”* Reese further explained that *“if I get sicker, then maybe they’ll take me more seriously.”* Participants reflected not only on their discontent with how these dynamics unfold in practice, but also on the inherent risks of condoning and operating within such institutionalized criteria. As Isabella puts it:*“it’s not ok that where it lands is that people who don’t show that they’re struggling, um, end up dead.”* Taken together, these accounts elucidate how weight-based thresholds operate not only as gatekeeping criteria but also as active producers of harm: they legitimize waiting for deterioration, normalize delayed intervention, and contribute to feelings of hopelessness and being undeserving of help.

Coming up Against Medical Categories and Labels*.* In a similar vein to BMI, weight, and other more recognized markers of severity, participants noted particular harms that emerge because of stringent diagnostic criteria and categories—particularly related to the DSM-5—which don’t always reflect the lived realities of those struggling with DEBs/EDs. *“[T]he approaches that are encouraged in the DSM-5 don’t account as much for the cultural and social realities…it’s too individual, I think is the bigger issue.” -Kimberly*. Participants did highlight some direct harms or difficulties which arose and impacted their ability to access care, for instance, Zara noted: *“[if] you don’t meet screening criteria…that really kind of can leave you sort of with a lifetime of not having services”.* Echoing the structural incongruities shaping access to care, Mía recounted waiting years for treatment, only to be deemed ineligible once she finally reached the front of the line: *“I didn’t meet the criteria, so they just said, okay, looks great, like, go back to your life.”* - Mía

Further, beyond accessing care alone, participants identified how service providers’ internalization of and overreliance on the DSM-V can result in care that is ill-fitting to what participants need, leaving them dissatisfied with the care received:

*“Even though she’s critical of the DSM as well, she’s also a psychologist… she’s internalized some sort of relationship to, sort of clinical categories and stuff like that… I’ve been a little bit let down with, I guess, what I perceive to be her willingness to engage with, with, kind of, the complexity of disordered eating in my life…”-Kimberly*

Some participants described being excluded from treatment when their experiences—such as binge eating or overeating—did not fit the dominant typologies of AN or BN. Because these diagnoses are positioned as quintessential types of EDs, participants whose experiences fell outside of them frequently encountered barriers to care.

*“I went to another program, and I had absolutely nothing for someone like me, someone who was a compulsive overeater or emotional overeater… if you’re bulimic or anorexic, there is definitely something out there for you, but even at the hospital, there was nothing for people like me. Nothing.”-Georgina*

Alex reflected a similar sentiment in their struggles with avoidant/restrictive food intake disorder (ARFID), highlighting how even disorders which fall within the DSM-V can remain unrecognized, resulting in them falling through the cracks and remaining undiagnosed after decades of struggling with their ED/DEBs.

*“I also struggle with ARFID…. nobody would have understood that that was what I was going through… It’s very frustrating, and, um, and I’ve never been diagnosed… I’m [age] years old, and I’ve been suffering for all this time.” -Alex [FG1]*

This also arose when participants felt that, during an assessment, the symptoms they were experiencing did not seem as troubling in that moment. By answering honestly about her symptoms, Valentina reflected on how failing to present with the “right” symptoms at a particular point in time could lead to being overlooked and unrecognized:

*“I went to an eating disorder specialist for an assessment, and at the at the time, the symptoms were not there, and things were going well, so I didn’t meet the criteria.”-Valentina*

When asked about the kinds of DEB/ED symptoms they’re struggling with, some participants felt compelled to explain or justify them in relation to formal diagnostic criteria. For example, Samuel described his experiences using DSM terminology, assuming this was necessary to “legitimize the data”: “*I figured for the data’s sake, like the the formal diagnosis DSM stuff would be somewhat helpful in legitimizing the data...”.* Highlighting one of the key points to come out of this thematic area, Samuel’s quote demonstrates how the need to ‘legitimize’ ‘verify’ or ‘categorize’ people with DEBs/EDs based on stringent criteria can silence peoples’ voices resulting in frustration with the fact that you cannot be heard if you are not speaking in biomedical and positivist terms that gatekeepers will understand. Later in his interview, Samuel further describes his frustration with discussing his ED/DEB symptoms with his family doctor, stating “*I’m almost convinced that she is more focused on formal diagnoses than anything”.* Again, highlighting that rigid criteria and diagnostic categories do not always align with or wholly represent the lived and living experiences of those with DEBs/EDs.

Some participants resisted conforming to DSM categories, underscoring a central point in this theme: the complexities and intersections of lived-experience cannot be reduced to rigid diagnostic criteria. Such reductionism can obscure lived-experiences. As Ben explained:

*“I don’t know whether there are discrete disorders that exist in people’s minds and brains and bodies. I believe it’s much more fluid, dimensional… I find it’s just very comfortable for me to say I experienced some symptoms that are typically associated with this, this and this, because then I can kind of construct my own sort of, like, description of my experience, because every experience is different” - Ben*

### “Nobody was worried about me…”: Being seen, misread, or overlooked

From participants’ perspectives, recognition of EDs/DEBs was shaped not only by institutional and medicalized criteria but also by sociocultural and relational contexts. Within this theme, two interrelated yet distinct narrative threads emerged. First, participants emphasized how recognition was mediated through *Norms of ‘Acceptability’ and Control*, whereby conformity to—or deviation from—normative expectations influenced visibility. For example, symptoms more readily pathologized were often recognized, whereas other behaviours could be overlooked or dismissed. Second, participants underscored the role of *Racialized and Cultural Dimensions of Recognition*, highlighting how race, ethnicity, and culture shape perceptions of who can be recognized as legitimately struggling with EDs/DEBs.

Norms of ‘Acceptability’ and Control. An important subtheme concerned how participants navigate dominant cultural norms of eating and body regulation, often by adhering to—or over-performing—gendered expectations before being perceived as having gone “too far”. Recognition was not shaped simply by the presence of EDs/DEBs alone, but rather by whether those behaviours remained legible within culturally sanctioned ideals of discipline, restraint, and control. ED/DEB symptoms aligning with normative femininity were frequently described by participants as being normalized—or even praised. Yet, similar behaviours remained pathologized only once they exceeded socially acceptable thresholds. In this sense, recognition was mediated less by divergence from norms than by when and how participants’ practices crossed an implicit line between acceptability and excess. Vivian explained how restriction was normalized and even praised, both in North American and Chinese cultural contexts, whereas purging was more pathologized in comparison:

*“I think restricting is almost so normal, again, in our culture and in my, like, in Chinese culture that, you know, ‘Oh I skipped a meal, no big deal.’ But when someone says like, ‘I made myself throw up to try and restrict my weight,’ like, okay that, that’s more… pathologized, in comparison.”*

This sociocultural framing positions restriction as socially acceptable and even commendable, while purging becomes a marker of “crossing the line,” thereby shaping whether behaviours are recognized as problematic.

Kimberly expanded on this idea, highlighting how women’s bodies are policed through narrow thresholds of acceptability. In her account, recognition of disorder was mediated by how closely one’s practices aligned with forms of control which are socioculturally endorsed:

*“[W]hether it’s bingeing, or, you know, restraint with anorexic categories or whatever it is, it’s just a problem when you’re slightly over the acceptable regulation of women’s bodies… anorexia and purging and bulimia have very different connotations in terms of your ability to control your body and yourself, and it’s like nobody wants unruly women’s bodies, right?”*

She noted the stark contrast in her family and friends’ responses to her own experiences, where restriction within a “normal” body size was praised, while purging elicited concern only from those who knew her intimately:

*“Nobody was worried about me, the people who were worried about it were people who knew that I was purging.”*

Adding further nuance, Rawiya highlighted how cultural pressures to remain thin could, in some contexts, override the perceived non-normativity of purging. She recounted:

*“[S]he purges after food, and her parents are proud, which is very sad because to them, they’re like, well, she’s purging, but she’s maintaining her weight. She looks good, so it doesn’t matter if that’s how she maintains her weight.”*

This account illustrates how the valorization of thinness can normalize—even condone—behaviours typically pathologized in other contexts. Here, purging was not recognized as harmful but rather reframed as an acceptable strategy for achieving and sustaining cultural ideals of body size.

These accounts show how dominant discourses of “acceptable” versus “unacceptable” control shape recognition of EDs/DEBs. Produced through intersecting biomedical, media, and gendered norms, restraint and self-discipline are valorized, while behaviours such as purging are marked as excessive or deviant. Restriction is often normalized or praised, whereas other behaviours are more readily pathologized. Recognition thus hinges not only on symptoms, but on alignment with gendered imaginaries of discipline and control, dynamics most pronounced among women.

Racialized and Cultural Dimensions of Recognition. Participants also highlighted sociocultural and relational factors that influence recognition, such as how race and ethnicity affect others’ perceptions of who is identified as having EDs/DEBs. Recognition was influenced not only by stereotypes about who “can” have DEBs/EDs but also by tensions between participants’ cultural backgrounds and Western biomedical views of mental health. For many, these conflicting frameworks meant that eating- or body-related distress was not easily acknowledged within their families or communities, and was often misunderstood or dismissed in clinical settings, deepening self-doubt about whether their experiences merited care.

Participants spoke about recognition being delayed—or outright denied—because of their racial/ethnic identity being in misalignment with dominant Western images of who has DEBs/EDs:

*“Let’s say I was white, like, I really think that people would have caught on a lot sooner that I had an eating disorder.”-Vivian*

Vivian’s account illustrates how being a person of colour rendered her ED unintelligible to providers compared to white patients, revealing racialized assumptions in clinical settings about who is seen as likely to struggle with EDs/DEBs. Further to this point, in the clinical context, Vivian reflects on race-based assumptions around BMI, which she viewed as being utilized to minimize her experience with an ED—ultimately impacting her ability to access help.

*“There were… a lot of comments like, yeah, you're young, you’re East Asian, and you’re thin… East Asian people… have a lower BMI threshold… I get where they’re coming from, but… it influenced the way… they perceived how much you need help.”* – Vivian

In some cultural contexts described by participants, DEBs/EDs were not widely recognized as legitimate illnesses. For instance, Rawiya reflects on the complexities of recognition within her own culture:

*“There was no awareness about mental health… in my culture, in Saudi Arabia, [EDs were] not a thing. The only thing they knew was depression… and even with depression, they think you could just knock yourself out of it. So… they were not aware it was an eating disorder… they thought I was being lazy.” – Rawiya*

She illustrates how mental health understandings vary across global contexts, leaving her struggles unrecognized and untreated. Rawiya described being labeled “lazy,” reflecting an individualized logic that blames those who struggle. Others likewise noted that cultural food practices made EDs seem unintelligible, resulting in minimal recognition from friends and family.

*“[I]n Chinese culture, eating disorders are not… culturally we… struggled with the opposite, which is like living through a famine. And food is so ingrained… when you say hi to someone in Cantonese, you don’t say hi, you say, ‘Oh, did you eat yet?’… I think the idea of an eating disorder was so foreign to them, they would never in a hundred years have thought [of an] eating disorder ever.” – Vivian*

Despite differences across contexts, participants also highlighted the pervasiveness of thin ideals globally. As Rawiya explained, *“I would say all cultures they really like people who are thin. So when someone who is not, they don’t really like to listen to them that much.”.* Here, Rawiya underscores that even amid cultural and geographic diversity, thin ideals persist across contexts. She draws attention to how individuals who do not conform to these ideals have their perspectives discounted, rendering their voices less likely to be heard.

For racialized participants, recognition was further shaped by the clash between cultural norms and their communities of origin and dominant Western ideals equating thinness with health and beauty, amidst encounters of racism. Maddi reflected:

*“Women of African descent who come to [Canada], it is only then that the eating disorder would kick in… because of their lived-experience of racism on arrival. Also, the way [Canada] thinks of bodies and the media… floods us with thin equals health and beauty.*

Maddi’s account highlights tensions shaping African women’s experiences after migrating to Western contexts. Exposure to anti-Black racism became embodied through disordered eating, while immersion in Western thin ideals intensified vulnerability. Together, these intersecting conditions shaped how EDs/DEBs emerged and were experienced within her community. Racialized and sociocultural norms constrained how EDs/DEBs were recognized and legitimized across family, community, and clinical contexts, influencing both external recognition and participants’ own processes of self-recognition—or its absence.

### “I just thought that it was me being fat and lazy…”: Navigating self-recognition

Recognition processes extended beyond social interactions to include whether individuals recognized themselves as having EDs/DEBs. Two interrelated threads emerged: *Conceptualizing* *the Problem as Disordered* and *Deciding to Access (or Avoid) Treatment*. The former reflects how participants made sense of their struggles, while the latter captures the often fraught decision to seek care, shaped by perceptions of worthiness and legitimacy. Participants described internalizing dominant cultural norms around eating, control, and body size and using these standards to assess their own experiences. This often led them to minimize or dismiss their behaviours as “normal”, “not serious enough”, or undeserving of care. Recognition was thus negotiated internally as well as interpersonally, shaping when—and whether—participants sought help.

Conceptualizing the Problem as Disordered. Many participants—directly relating back to the theme of “You have to present a certain way…”: Struggles for Legitimacy—internalized ideas of weight and BMI as indicators to *themselves* as to how they are doing with their DEBs/ED, sometimes relating back to the notion of ‘losing control’, and often playing a role in their determination of their need for treatment—seen in the theme to follow. Pushing this idea further, this subtheme highlights how markers of legitimacy and severity influence how participants perceive their own difficulties:

*“It’s been in the past year that I’ve felt less and less in control of watching my weight go up… I just don’t seem to have any control over it anymore.” -Cynthia*

Weight, BMI, and other stringent criteria play a role in peoples’ self-conceptualization of whether or not what they are doing is disordered or problematic, and also whether or not it is volitional. For example, in reflecting on the realization that her experiences aligned with disordered eating, Rawiya explained: *“I just thought that it was me being fat and lazy, but then it came about that there is a bigger problem there.’* Her account underscores the complexity of self-recognition in identifying struggles with DEBs/EDs while living in a larger body.

Participants also emphasized that sociocultural mediators intersect with cultural ideas and Western norms to influence how they perceive themselves:

*“I’ve seen so many videos online about how like brown girls especially are usually like ‘skinny fat’ … we’re not really seen as skinny people, and I think that played a role in how I looked at my body…”-Aanya*

Participants, at times, became their own gatekeepers, often cognizant of not wanting to ‘occupy space they shouldn’t’ or co-opt the language of eating disorders when they haven’t been diagnosed. This is predominantly described in a way that implies participants utilize caution as not to ‘co-opt’ the gatekept language of ‘eating disorder’:

*“I don’t have eating disorders. I have disordered eating. Yeah, I think that’s important to distinguish because eating disorders are very serious. It is different.” -Grace*

*Deciding to Access (or avoid) Treatment.* Participants described the internal reasoning shaping decisions to access—or avoid—treatment. Building on the self-understandings captured in *Conceptualizing the Problem as Disordered*, the theme *Deciding to Access or Avoid Treatment* examines how these perceptions translated into care-related choices. Participants navigated barriers such as feeling unworthy of support, anticipating judgment, and fearing they were “taking up space” better reserved for others. At the same time, some identified facilitators that prompted treatment-seeking, including prior positive encounters with health professionals and reaching self-recognized thresholds of distress or loss of control.

Participants frequently perceived their struggles as illegitimate in the eyes of others, shaping how they assessed their own worthiness for care. Reflecting on this process, Kimberly explained:

*“I’m concerned that people don’t read me as having a valid relationship to disordered eating…I worry, because I know the extremes… for example, people who have to navigate the outward perception that somebody obviously has disordered eating experience it way differently than me.”*

Kimberly further iterated this tension, drawing a comparison between entering a space designated for Queer People of Colour (QPOC) as a White person:

*“I would feel pretty gutted if I walked into the space as the only person who was not read as having an unruly body… I feel like I’m taking up space where I shouldn’t be. Like, I’m not going to go to a QPOC event and be like, I have a right to this space, right?”*

To Kimberly, entering ED-specific spaces felt like taking up space where she didn’t feel she belonged.

For others, the decision to access treatment was linked to self-reflective indicators, particularly feelings of being “out of control”—in direct relation to the aforementioned theme of conceptualizing the problem as disorder—or changes in weight. When describing coming to the decision to access care, Emma shared how weight gain shaped her decision:

*“…I wanted to access treatment because I had a significant amount of weight gain and, because of my disordered eating, I felt like I just didn’t know how to eat properly and be healthy.”*

This account shows how weight and other institutionally sanctioned markers of “severity” shape self-perception and care-seeking. Participants often used weight—an indicator shaped by biomedical and cultural norms—to evaluate health status. For some, this silenced concerns and minimized distress; for others, weight change legitimized symptoms and prompted help-seeking. Thus, sanctioned severity markers both constrained and enabled access to care by shaping when individuals perceived themselves as warranting support.

Notably, positive prior encounters with service providers were also powerful facilitators of help-seeking. Grace reflected: *“Having positive experience of previous professionals definitely helped me make that decision. Knowing that I had received a lot of great support from my psychiatrist made me a lot more open to receiving support from other types of professionals.”* Participants’ treatment decisions reflected negotiations of legitimacy, self-worth, and social comparison: feelings of unworthiness constrained access, while supportive relationships and reaching perceived distress thresholds encouraged care-seeking.

## Discussion

The findings reveal three interconnected dimensions of recognition and visibility in EDs/DEBs. First, *“You have to present a certain way…”: Struggles for Legitimacy* captured how access to care hinged on demonstrating visible indicators such as weight loss, BMI thresholds, or DSM-5 criteria. Participants who did not meet these benchmarks were often denied services, leaving their struggles overlooked or dismissed.

Second, “Nobody was worried about me…”: Being Seen, Misread, or Overlooked showed how broader norms and power relations shape legitimacy in treatment contexts. Participants, particularly BIPOC individuals, described being misread or dismissed through implicit racism and microaggressions. Dominant ideals around body size and “acceptable” disordered behaviours further shaped whose distress was recognized, rendering some struggles invisible while others were more readily perceived as warranting care.

Finally, *“I just thought that it was me being fat and lazy…”: Navigating Self-Recognition* captured how participants came to understand their experiences as disordered and how this shaped help-seeking. Messaging around weight, BMI, and diagnostic thresholds intersected with sociocultural and institutional norms to determine whether struggles were seen as “serious enough.” Recognition thus operated both externally and internally, shaping access to care and recovery trajectories.

### Institutionalized criteria as exclusionary

BMI thresholds and other physical “severity” indicators act as exclusionary gatekeepers, disproportionately affecting those already underrepresented in care. These measures perpetuate stigma, underestimate prevalence in marginalized groups, and bias eligibility for treatment or research participation, exacerbating inequities [27]. Individuals with normal or higher BMI, or a history of obesity, are often identified later, resulting in poorer prognoses given the importance of timely treatment [53]. Exclusions compounded by weight stigma and clinician bias [54,55] facilitate what Dhairyawan [19] terms *medical silencing*, where patient testimony is dismissed for not fitting biomedical paradigms. Such silencing produces epistemic and material harms, including delayed or denied care, rendering recognition uneven across individual, relational, and systemic levels. Institutionally, BMI cut-offs and diagnostic thresholds gatekeep recognition, leaving many inadequately served and paradoxically forced to deteriorate further before accessing care [32].

We contend that over-reliance on physical indicators marginalizes mental and emotional dimensions of health [32]. When biomedical criteria contradict and supersede lived-experience, they produce testimonial injustice [20] and enact epistemic violence that can culminate into medical silencing [19]. The stakes are high: as Isabella noted, *“people who don’t show that they’re struggling, um, end up dead.”.* Even those fitting normative understandings are not exempt—Reese, for example, despite struggling with AN, was excluded from both high- and low-intensity treatment due to BMI thresholds—she fell between the cracks, remaining ‘too sick’ for some treatment options yet ‘not sick enough’ for others.

Participants repeatedly described needing to “get worse before they could get better,” echoing findings that weight loss paradoxically became the most effective way to access recovery [32]. This directly challenges stereotypes of *volitional stigma*—that individuals “want to be sick” [56,57]. Rather, systemic reliance on exclusionary criteria positions deterioration as a condition of entry—requiring people with DEBs/EDs to *prove* a certain level of sickness before accessing care, thereby reproducing harm within the very systems meant to support them. We therefore call for critical reconsideration of weight, BMI, and DSM-5 thresholds. While not arguing for their abandonment, their use must acknowledge the epistemic and material harms they produce and re-examine dominant notions of “severity.” Ironically, the very preoccupation with weight that treatment seeks to reduce is simultaneously reinforced by such institutional practices [58].

### Self-recognition and hermeneutic injustice

Dominant discourses often pathologize people with EDs/DEBs as being “in denial” of their disorder. What this overlooks is the lack of interpretive frameworks available for individuals to make sense of disordered eating. This produces what Fricker [20] terms *hermeneutic injustice*—where people lack the conceptual resources to understand their own struggles. Our findings illustrate this dynamic: participants described carefully avoiding the “co-opting” of eating disorder terminology or rejecting DSM-5 categories, viewing themselves as unworthy or illegitimate in claiming such labels. While existing literature highlights ambivalence toward recovery, denial of severity, or outright denial of illness [59], such discourse risks misplacing blame. A critical question emerges: *How can we expect people to see themselves as having an eating disorder when the very criteria by which recognition is granted do not align with their lived realities?*

### Shifting the collective imagination

The collective imagination of what it means to struggle with DEBs/EDs remains fraught with stereotypes, enacted across individual, social, and institutional levels [23,27]. These imaginaries, alongside “You have to present a certain way…”: Struggles for Legitimacy, extend beyond provider–patient interactions to shape systemic structures of recognition. While individual acts of resistance to such criteria are important, they alone cannot shift the broader social re-imagination of eating disorders and disordered eating. Systemic and individual change are therefore both necessary to re-imagine how DEBs/EDs are experienced.

It is not only “skinny white women” who experience EDs; yet dominant imaginaries erase those who fall outside this stereotype [24,26,60], leaving many to endure—as described by Zara—*“a lifetime without accessing services”*. Remediating these disservices requires more expansive representation across media, literature, and public discourse. Those outside the SWAG stereotype do not lack EDs—they remain invisible within existing Western biomedical framings. These dynamics unfold across multiple, intersecting identities, where some facets confer privilege and others marginalization, always entangled with temporal and spatial contexts.

### Methodological contributions and limitations

This paper makes several methodological contributions by integrating intersectionality and narrative inquiry, positioning intersectionality [21] as both theory and paradigm [44] shaping how stories are elicited, interpreted, and analyzed. In this way, findings further demonstrate how intersectional narratives operate across individual, relational, clinical, and sociocultural contexts without collapsing analytic levels [61], sharpening attention to the politics of recognition and visibility.

This study is not without limitations. To preserve confidentiality—particularly given recruitment through a single organization—participant demographic data were aggregated in ways that may obscure some intersectional nuance, such as identification with multiple racial groups. While this limits the reader’s ability to fully trace layered identities, these complexities were central to data collection, analysis, and theme development. As highlighted in the findings, self-recognition often functions as a prerequisite for participation, potentially leaving some forms of distress unrepresented.

## Conclusion

This study contributes to scholarship on recognition and visibility in EDs/DEBs by tracing how struggles for legitimacy, sociocultural and relational mechanisms, and processes of self-recognition intersect to shape experiences of disorder and care. We demonstrate how rigid diagnostic criteria and markers of severity silence individuals whose suffering does not align with “typical” presentations, often delaying access until significant deterioration occurs. In doing so, the findings illuminate how epistemic violence and related forms of medical silencing operate in the lives of people with EDs/DEBs [19].

For health systems and clinicians, these findings underscore the need to re-examine BMI thresholds, diagnostic categories, and assumptions about normative presentations, as discounting patient testimony produces both epistemic and material harms. We argue for weight-neutral, trauma-informed approaches that better attend to intersectional identities and lived complexity. Methodologically, this study demonstrates the value of intersectionality as both paradigm and practice. Rather than privileging reduction or thematic simplification, we foreground overlap, entanglement, and multiplicity as central to lived-experience, reconceptualizing qualitative rigour as sustained engagement with complexity. Recognition remains unevenly distributed, shaped by what Fricker [20] terms the “collective social imagination,” through which identity power silences certain voices. Attending to these dynamics is essential for building health systems capable of recognizing those whose struggles remain unseen.

## Supporting information

S1 Table Sample Demographic Characteristics (N = 23). (DOCX)

S1 File COREQ Checklist. COREQ (COnsolidated criteria for REporting Qualitative research) Checklist.
(PDF)
