# Supplementary material for: “I feel like there’s going to have to be catastrophe before I am seen:..and that is going to be devastating”: Visibility, recognition, and medical silencing in eating disorders and disordered eating
Source: PLOS Ment Health. 2026 Jun 17;3(6):e0000634. doi: 10.1371/journal.pmen.0000634 (PMC13274819; doi:10.1371/journal.pmen.0000634)
Supplement: S1 Table — (DOCX) [file pmen.0000634.s001.docx]

**S1 Table.** Sample Demographic Characteristics (N=23)

| **Characteristics** | **Category** | **Frequency (n)** | **Percentage (%)** |
| --- | --- | --- | --- |
| **Age** | 18-24 | 3 | 13 |
|  | 25-34 | 7 | 30 |
|  | 35-44 | 3 | 13 |
|  | 45-54 | 5 | 22 |
|  | 55-64 | 3 | 13 |
|  | 65 and Over | 2 | 9 |
| **Gender Identity*** | Female | 15 | 65 |
|  | Non-Binary | 2 | 9 |
|  | Transgender | 2 | 9 |
|  | Male | 4 | 17 |
|  | Other | 1 | 4 |
| **Sexuality*** | Bisexual | 4 | 17 |
|  | Heterosexual | 14 | 61 |
|  | Lesbian | 2 | 9 |
|  | Queer | 3 | 13 |
|  | Pansexual | 1 | 4 |
|  | Demisexual | 1 | 4 |
|  | Prefer not to answer | 1 | 4 |
| **Racial or Ethnic Group*** | Black | 1 | 4 |
|  | Chinese | 3 | 13 |
|  | Latin American | 4 | 17 |
|  | Middle Eastern | 1 | 4 |
|  | Métis | 1 | 4 |
|  | South Asian | 2 | 9 |
|  | White | 12 | 52 |
|  | Other | 1 | 4 |
| **Disability or Chronic Condition*** | Learning Disability | 6 | 26 |
|  | Visually Impaired or Blind | 3 | 13 |
|  | Mental Health Condition | 13 | 57 |
|  | Mobility-related Disability | 4 | 17 |
|  | Speech-related Disability | 1 | 4 |
|  | Other | 4 | 17 |
|  | Prefer not to Answer | 3 | 13 |
| **Annual Household Income** | Under $30,000 | 5 | 22 |
|  | $30,000-$49,999 | 4 | 17 |
|  | $50,000-$74,999 | 2 | 9 |
|  | $75,000-$99,999 | 2 | 9 |
|  | $100,000-$149,999 | 4 | 17 |
|  | $150,000 or more | 2 | 9 |
|  | Prefer not to Answer | 4 | 17 |
| **Employment Status*** | Unable to work (Disability) | 5 | 22 |
|  | Student | 5 | 22 |
|  | Employed Part-Time | 7 | 30 |
|  | Employed Full-Time | 7 | 30 |
|  | Retired | 3 | 13 |
|  | Unemployed | 2 | 9 |
|  | Other | 1 | 4 |

*Note: the sum of some identity categories does not equal that of the sample size, as participants were able to select multiple options.
